# Surgical treatment in non-small cell lung cancer with pulmonary oligometastasis

**DOI:** 10.1186/s12957-017-1105-8

**Published:** 2017-02-02

**Authors:** Jinyuan He, Yun Li, Jun An, Liu Hu, Junhang Zhang

**Affiliations:** Department of Cardiothoracic Surgery, The 3rd Affiliated Hospital of Sun Yat-sen University, Guangzhou, China

**Keywords:** Non-small cell lung cancer, Oligometastasis, Oligo-recurrence, Sync-oligometastases

## Abstract

**Background:**

Previous studies have demonstrated survival benefits for local treatment in solitary metastatic non-small cell lung cancer (NSCLC).This study aimed to investigate the effect of local surgery for NSCLC with pulmonary oligometastasis.

**Methods:**

This study included 21 patients of NSCLC with pulmonary oligometastasis between January 2003 and December 2013, which were divided into two groups, group A (11 cases) for local surgery and group B (10 cases) for systematic chemotherapy, compared the median survival time (MST) and 5-year survival rate between the two groups, and analyzed the impact of the pathological types, the TNM and pN stage of primary tumor, the site, and the mode and number of oligometastatic nodule on group A.

**Results:**

The MST of group A and B were 37 and 11.6 months respectively, 5-year survival rates were 18.2 and 9.1% respectively (*p* < 0.05). Patients with single nodule, oligo-recurrence, primary tumor of pN0, TNM stage I or II obtained higher survival rate than those with multiple nodules, sync-oligometastases, pN1-2, stage III or IV in group A (*p* < 0.05). There was no significant survival time difference among pathological types of primary tumor and oligometastatic site (*p* > 0.05).

**Conclusion:**

Local surgery significantly prolonged the overall survival time and 5-year survival rate of primary NSCLC with pulmonary oligometastasis.

## Background

Primary lung cancer is one of the most common malignant tumors and is still the leading cause of cancer-related death in many countries [1, 2]. Approximately 220,000 new cases and 150,000 related deaths are reported each year in the USA [3]. In China, the incidence of lung cancer is increasing every year, and mortality is about 0.03%. In clinics, about 80–85% of primary lung cancers are non-small cell lung cancer (NSCLC). Surgical radical resection is still the most effective treatment for early stages. However, 20–50% patients present with metastasis, the prognosis is poor. The median survival time is about 8–11 months [4]. Approximately 7% of patients with advanced metastatic NSCLC have a solitary metastasis after full evaluation [5]. These solitary metastases include sync-oligometastatic or oligo-recurrent satellite nodules in different pulmonary lobes and solitary extrapulmonary metastases. Hellman and Weichselbaum [6] proposed the term “oligometastasis,” which was revised by Niibe et al in 2006 [7], to describe this restricted local regional tumor load. It was a less biologically aggressive tumor stage with a metastatic number less than five and metastatic site limited to one single organ, which remain stable over time. Many previous studies have confirmed the presence of oligometastasis in NSCLC [8–10] and shown the significant effects of local treatment in improving the overall survival time. However, most of these studies were focused on extrapulmonary oligometastasis, rarely on pulmonary oligometastasis.

In the present study, we retrospectively analyzed the maintained database of patients with pulmonary oligometastasis of NSCLC in our hospital between 2003 and 2013, compared the survival differences between local surgery and systematic chemotherapy, and investigated the effect of local surgery on overall survival time, median survival time, and 5-year survival rate.

## Methods

### Patients

We retrospectively identified 21 patients of primary non-small cell lung cancer with pulmonary oligometastasis in the maintained database (His System) that were followed up between January 2003 and December 2013 at the third affiliated hospital of Sun Yat-sen University. The inclusive criteria were as follows: (a) All patients undergone complete primary tumor resection for NSCLC; (b) There were one to three pulmonary nodules in addition of the primary tumor, including sync-oligometastatic and oligo-recurrent nodules, which were technically resectable; (c) No distant extrapulmonary metastases were found. To differentiate pulmonary oligometastasis from second primary lung cancer, we used the Martini and Melamed criteria [11], which were modified by Antakli et al [12] and Girard et al [13]. In this study, “oligometastases” were defined as limited number (one to five) of pulmonary nodules which could be controlled by local surgery. Sync-oligometastases were defined as pulmonary nodules that were discovered simultaneously or in 6 months after NSCLC were diagnosed, over 6 months was called oligo-recurrence.

This study was approved by the Ethics Committee for Clinical Research of the third affiliated hospital of Sun Yat-sen University.

### Treatment technique

The 21 inclusive patients were divided into two groups, the surgery group and the chemotherapy group. Routine preoperative checkups were done, including laboratory assessments of the blood cell count, serum chemistry, the serum tumor markers (CYFRA and NSE), cardiopulmonary function, and thoracic computed tomography. The surgical approach included pulmonary wedge resection and lobectomy, according to the patients’ general condition. For sync-oligometastases, the time between primary tumor radical resection and metastatic nodules resection should be less than 2 months.

Either radiotherapy or chemotherapy was prohibited before local metastatectomy, with the exception of adjuvant chemotherapy; after primary radical resection, oligo-recurrence was detected. All patients in the surgery group accepted adjuvant chemotherapy or dendritic cell-cytokine-induced killer cell (DC-CIK) immunotherapy after metastatectomy.

The patients in the chemotherapy group accorded with the inclusive criteria, however, could not tolerate operation because of older age, poor physical condition, or other reasons.

### Follow-up

The overall follow-up time was 12 to 72 months, and the visits were scheduled at 3-month interval for the first 3 years, 6-month interval for the remaining years. At each visit, physical examination, laboratory assessments of the blood cell count, serum chemistry and the serum tumor markers (NSE and CYFRA), and chest X-rays were performed. In addition, thoracic CT and bone scans were done when clinically indicated.

### Statistical analyses

Statistical analysis was performed using SPSS15.1 (SPSS Inc, Chicago, IL). The Kaplan-Meier test was used to analyze overall survival and 5-year survival rate, which were calculated from the date of primary radical resection of NSCLC to death or the last follow-up for patients who were still alive. Univariate analysis between groups was done by the log-rank test. A two-sided *p* value of <0.05 was considered to be statistically significant.

## Results

### Patient characteristics

The surgery group included eleven patients (Table 1 shows patients’ characteristics). The media age was 56.4 years, ranged from 41 to 75 years, including six males and five females. The diameter of primary tumor was 1.5–7.1 cm, and the number of metastatic nodules was 1–3. Ipsilateral pulmonary oligometastases were found in four patients, and contralateral in other seven patients. Sync-oligometastases and oligo-recurrence were found in three and eight patients, respectively.

**Table 1:** Clinical characteristics and survival for the surgery group (*n* = 11)

Patients	Sex	Age (years)	Size (cm)	pN	TNM stage	Histology	Oligometastatic			Surgical approach	Outcome	Survival
			of primary tumor				Mode	Number	Site			(Months)
1	M	61	1.6	0	IA	Ad	R	1	IL	PWR	Dead	69
2	F	45	1.5	0	IA	Sq	R	1	IL	PWR	Alive	71
3	F	71	2.8	2	IV	Ad	S	2	CL	PWR	Dead	21
4	M	44	3.2	1	IIA	Ad	R	2	CL	PWR	Alive	37
5	F	59	5.4	1	IV	Ad	S	2	IL	PWR	Dead	29
6	M	63	1.9	1	IIA	Ad	R	1	IL	PWR	Alive	42
7	F	52	2.3	0	IA	Ad	R	1	CL	PWR	Dead	51
8	M	75	7.1	2	IIIA	Lc	R	2	CL	PWR	Dead	15
9	M	47	2.2	0	IA	Ad	R	1	CL	PWR	Alive	41
10	F	41	6.4	1	IIB	Ad	R	3	CL	LO	Dead	17
11	M	62	4.5	2	IV	Sq	S	1	CL	PWR	Dead	19

Abbreviations: *M* male, *F* female, *Ad* adenocarcinoma, *Sq* squamous cell carcinoma, *Lc* large cell carcinoma, *R* oligo-recurrence, *S* sync-oligometastasis, *IL* ipsilateral lung, *CL* contralateral lung, *PWR* pulmonary wedge resection, *LO* lobectomy

Pulmonary wedge resection was performed in ten cases, six of which only had one metastatic nodule. Lobectomy was done only in one patient, with three oligometastatic nodules locating in the same lobe. The average time interval between sync-oligometastatic resection and primary tumor radical resection was 41.4 days. No serious postoperative complications were found among patients. The postoperative pathological diagnosis showed seven cases that were adenocarcinoma, three squamous cell carcinoma, and one large cell carcinoma.

Postoperative adjuvant chemotherapy was done among the eleven patients, eight of whom finished four courses, three accepted DC-CIK immunotherapy after one or two courses, because of serious chemotherapy blood toxicity. The chemotherapy group included ten patients, with the median age of 60 years, six of which were males four were females.

### Survival

**Fig. 1:**
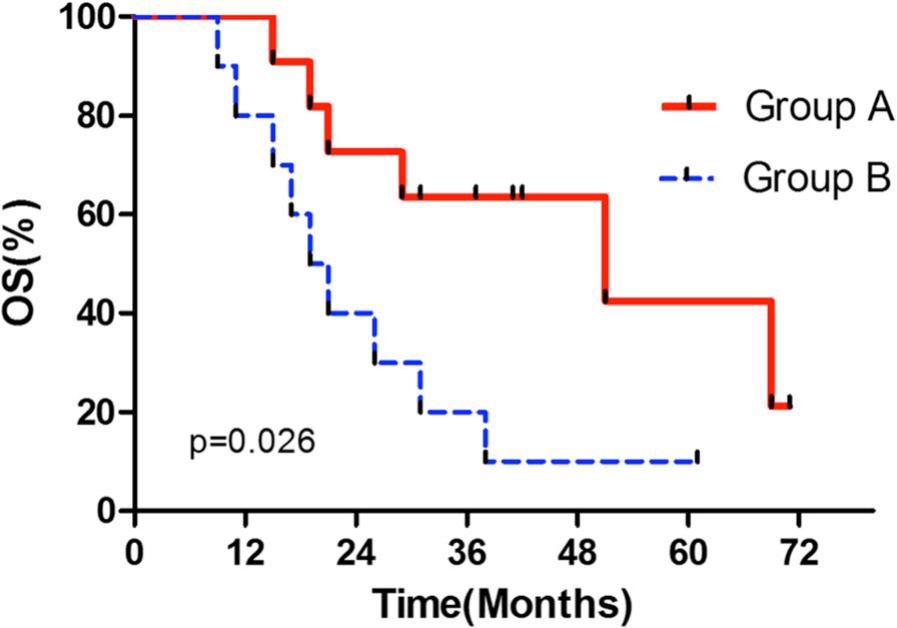
The Kaplan-Meier curve of overall survival time for group A and B

All patients were followed up for 15–72 months; the median follow-up time was 37.5 months. In the local surgery group, four patients (36.4%) were alive with stable disease at the time of the analysis, the remaining died of specific lung cancer progression during the follow-up period. The overall survival time (OS) ranged from 19 to 71 months for the surgery group, and median survival time (MST) was 37 months. The OS for chemotherapy group was 9–61 months, while MST was 11.6 months. The overall 5-year survival rate for the surgery and chemotherapy group was 18.2 and 9.1% respectively. The OS and 5-year survival rate of the surgery group were significantly higher than the chemotherapy group (*p* < 0.05). Figure 1 shows the Kaplan-Meier curve for the OS of the two groups.

Table 2 shows the univariate analysis of the potential factors for overall survival of the surgery group. The sex, age, the primary tumor site and oligometastatic site (ipsilateral or contralateral), and histological subtypes showed no statistical significant differences to survival (*p* > 0.05). However, in lower pN and TNM stage, single and oligo-recurrence were associated with better survival (*p* < 0.05).

**Table 2:** Univariate analysis of predictive factors for OS of the surgical group (*n* = 11)

Predictive factors	Patients	5-year OS (%)	Log-rank *p* value
Gender			
Male	6	16.7	
Female	5	20.0	0.956
Age (years)			
>50	7	14.3	
≤50	4	25.0	0.176
Histological subtypes of primary tumor			
Adenocarcinoma	8	12.5	
Non-adenocarcinoma	3	33.3	0.855
Oligometastatic site			
Ipsilateral lung	4	50.0	
Contralateral lung	7	0.00	0.104
Oligometastatic mode			
Sync-oligometastasis	3	0.00	
Oligo-recurrence	8	25.0	*0.012*
Number of oligometastatic nodules			
One	6	33.3	
Two or Three	5	0.00	*0.040*
pN of primary tumor			
N0	4	50.0	
N1 or N2	7	0.00	*0.038*
TNM stage of primary tumor			
I or II	7	28.6	
III or IV	4	0.00	*0.0004*

Italicized figures represent statistically significant difference

## Discussion

Although local treatment in metastatic non-small cell lung cancer is still controversial, many previous research [10, 14–16] have reported parts of selective patients that benefited from it, but radical resection of the primary tumor was necessary. Many studies [6, 17–19] also demonstrated local resection of the metastatic lesion that improved the survival time. The 5-year survival rate reached 15% after surgical resection of the solitary brain metastasis, and 25% for solitary adrenal metastasis after adrenalectomy. Tokujiro Yano et al [20] reported that the median FPS and overall survival time were 12.1 months and 13.5 months after local treatment for sync-oligometastasis of NSCLC, but only a small part of patients benefited. However, patients with oligo-recurrent effectively benefited, whose median FPS reached 20 months. Our present study was consistent with their viewpoint. The OS for oligo-recurrence was 41.5 months compaired with 21 months for sync-oligometastasis, which was significantly different (*p* = 0.012).

Given the favorable results of local treatments for oligometastatic disease, new treatment strategies based on new diagnostic criteria and aggressive treatment options must be considered. The study from Postmus et al [21] shown 5-year survival rate that was about 28% for pulmonary oligometastatic satellite nodules resection, which was much more favorable than the patients who received palliative systemic chemotherapy. Kozower et al [18] reported 5-year survival rate that was 13% after resection of ipsilateral pulmonary oligometastasis. Voltolini et al [22] analyzed long-term survival rates after complete surgical resection of synchronous multiple lung cancers, both ipsilaterally (*n* = 27) and contralaterally (*n* = 28), the 5-year survival rate was not significantly different between the two groups, reported to be 27 and 43% respectively. They further demonstrated lymph node metastases which were important parameters influencing prognosis. In a study of 66 patients of synchronous bilateral pulmonary oligometastasis, OS and 5-year survival rate were reported to be 25.4 months and 38% respectively after simultaneously resection of bilateral metastastic nodules [23]. The recent study from Endo et al [24] concluded that Clinical T1-2N0-1 NSCLC with oligometastatic lesion was a good candidate for surgical resection, 5-year survival rate of about 40% can be expected. They also demonstrated that it was important that the indication was limited to intrathoracic clinical stage I or II. Approximately 60% of such patients could undergo complete resection of both the primary NSCLC and a true metastasis, and benefited.

Despite the survival time that varies from each other, we conclude from the above literatures that local treatment does improve parts of selective patients with oligometastatic NSCLC and make them benefit. This is consistent with our study. The median survival time and 5-year survival rate in the surgical group were 37 months and 18.2%, respectively, which were significantly better than systematic chemotherapy. We also conclude from our study that patients with lower pN and TNM stage, single and oligo-recurrence were associated with better survival. These factors can be regarded as prognostic factors.

Mortality rates were reported between 0 and 2.5% in pulmonary metastasectomy for extrapulmonary primary tumors [25]. Thus, it can be expected that a sequential approach for NSCLC and oligometastatic disease can be done with a comparable low mortality rate. However, local oligometastasectomy after radical resection of NSCLC could be done only when patients can tolerate operation after total evaluation; otherwise, chemotherapy is the only choice. In this study, ten exclusive patients underwent chemotherapy because of older age, poor physical condition, or other reasons, with MST of 11.6 months, and died of tumor progression. To our experience, the preferred surgical approach is pulmonary wedge resection and segmentectomy; lobectomy is not recommended because of relatively higher operation risk and greater trauma. Only one patient underwent lobectomy in our study, postoperative recovery time is relatively longer than that in primary tumor radical resection. For sync-oligometastases, segmentectomy or wedge resection on the contrary side should be performed first if the primary tumor requires a lobectomy or bilobectomy.

## Conclusions

The study of local surgery in non-small cell lung cancer with pulmonary oligometastasis is rare. For these patients, our present study have demonstrated that the median survival time and overall 5-year survival rate reached 37 months and 18.2% respectively after pulmonary oligometastasectomy. NSCLC patients with pulmonary oligometastasis could benefit from local surgery.

## Abbreviations

MST: Median survival time
NSCLC: Non-small cell lung cancer
OS: Overall survival time
